# Tirzepatide-Induced Gastrointestinal Manifestations: A Systematic Review and Meta-Analysis

**DOI:** 10.7759/cureus.46091

**Published:** 2023-09-27

**Authors:** Hani Raka Karrar, Mahmoud Ismail Nouh, Yousef Ismail Nouh, Mawadah Ismaeel Nouh, Adel S Khan Alhindi, Yousef Hassan Hemeq, Abdullah Mohammed Aljameeli, Jameel Abdullah Aljuaid, Saleh Jabbar Alzahrani, Abdullah Abdulrahman Alsatami, Mona Ali Alkredees, Abdulrahman Olayan Almuqati, Sobhia N Abanmi, Amal Mueidh Alshehri

**Affiliations:** 1 Pharmaceutical Care, Dr. Samir Abbas Hospital, Jeddah, SAU; 2 Medicine, Ibn Sina National College for Medical Studies, Jeddah, SAU; 3 Medicine, King Saud Bin Abdulaziz University for Health Sciences, Jeddah, SAU; 4 Medicine and Surgery, Ministry of Health, Taif, SAU; 5 Emergency, Herra Hospital, Makkah, SAU; 6 Pharmaceutical Care, Aljahra Hospital, Aljahra, KWT; 7 Pharmaceutical Care, Health Affairs, Taif, SAU; 8 Pharmaceutical Care, Prince Mohammed Bin Abdulaziz Hospital, Riyadh, SAU; 9 Pharmaceutical Care, Armed Forces Hospital Southern Region, Khamis Mushait, SAU; 10 Pharmaceutical Care, Shaqra General Hospital, Shaqra, SAU; 11 Medicine, Majmaah University, Riyadh, SAU; 12 Pharmacy, Princess Nourah Bint Abdul Rahman University, Riyadh, SAU

**Keywords:** gip, glp1ras, type 2 diabetes, gastrointestinal manifestations, tirzepatide

## Abstract

This systematic review and meta-analysis determine how frequently and how seriously gastrointestinal manifestations affect people with type 2 diabetes mellitus on tirzepatide. Tirzepatide is a recently developed drug that attempts to enhance type 2 diabetics' ability to regulate their blood sugar levels and promote weight reduction. Despite its potential benefits, clinical trials have revealed that the medication may lead to gastrointestinal side effects, including nausea, vomiting, decreased appetite, dyspepsia, constipation, and diarrhea. These side effects may negatively affect the drug's efficacy and patient tolerance. A comprehensive search of electronic databases such as PubMed, Web of Science, and Cochrane Library, was conducted to find pertinent studies reporting on the frequency and severity of gastrointestinal symptoms in type 2 diabetes patients receiving tirzepatide. This systematic review follows the Preferred Reporting Items for Systematic Review and Meta-Analysis (PRISMA) guidelines. Study selection, data extraction, and quality assessment were performed. Six randomized controlled trials with a total of 4,586 patients were included. Most patients received tirzepatide to regulate their blood sugar levels and promote weight reduction, and the comparators were placebo, glucagon-like peptide one receptor agonists drugs, and insulin degludec. The dose of tirzepatide was 5mg, 10mg, and 15mg weekly. The incidence rate of nausea in patients who receive tirzepatide was 20.43%, while the incidence rate in the comparators was 10.47%, and it was significantly higher in the tirzepatide arm than in the comparators (RR, 2.90; 95% CI, 1.89 to 4.44; P ≤ 0.00001). The incidence rate of vomiting in patients who receive tirzepatide was 9.05%, while the rate in the comparators was 4.86%, and it was significantly higher in the tirzepatide arm than in the comparators (RR, 2.69; 95% CI, 1.67 to 4.36; P ≤ 0.0001). The incidence rate of constipation in patients who receive tirzepatide was 2.54%, while the rate in the comparators was 0.856%, and it was significantly higher in the tirzepatide arm than in the comparators (RR, 3.08; 95% CI, 1.83 to 5.20; P ≤ 0.0001). The incidence rate of decreased appetite in patients who receive tirzepatide was 9.64%, while the rate in the comparators was 2.88%, and it was significantly higher in the tirzepatide arm than in the comparators (RR, 5.04; 95% CI, 3.01 to 8.45; P ≤ 0.00001). The incidence rate of diarrhea in patients who receive tirzepatide was 16.24%, while the rate in the comparators was 8.63%, and it was significantly higher in the tirzepatide arm than in the comparators (RR, 2.07; 95% CI, 1.60 to 2.68; P ≤ 0.00001). The incidence rate of dyspepsia in patients who receive tirzepatide was 7.13%, while the rate in the comparators was 3.31%, and it was significantly higher in the tirzepatide arm than in the comparators (RR, 2.52; 95% CI, 1.58 to 4.01; P ≤ 0.0001). Tirzepatide usage is linked to a significant prevalence of gastrointestinal symptoms, including nausea, constipation, decreased appetite, dyspepsia, diarrhea, and vomiting, in people with type 2 diabetes. These findings may influence clinical decision-making and patient counseling on the use of tirzepatide and have significant implications for the medication's tolerance and efficacy. To find ways to reduce these negative effects and improve therapy for type 2 diabetes patients, more research is required.

## Introduction and background

Type 2 diabetes mellitus is a complicated metabolic illness that not only causes hyperglycemia but is also accompanied by other metabolic issues including insulin resistance, decreased insulin secretion, hypertension, dyslipidemia, and more. As of 2019, there are approximately 463 million individuals worldwide who have diabetes mellitus, and by 2045, there are expected to be 700 million [1-3]. Numerous risk factors, like as genetic history, an unhealthy lifestyle, and obesity, might raise the chance of type 2 diabetes. It is thought that obesity is a powerful motivator [4]. Cardiovascular disease risk will rise with poor treatment of type 2 diabetes and obesity. There is evidence supporting the role of obesity in the development of type 2 diabetes and cardiovascular disease as well as the link between weight loss and a sustained reduction in cardiovascular risk [5]. Thus, optimum type 2 diabetes treatment should be effective in reducing glucose as well as encouraging weight reduction, both of which have been shown to have positive effects on the cardiovascular system and minimal risk of side effects [6]. As a result of its impressive glycemic control, helpful weight reduction, and positive cardiac outcomes, glucagon-like peptide one receptor agonists are currently recommended for treatment for type 2 diabetes patients [7]. Along with glucagon-like peptide one receptor agonists, glucose-dependent insulinotropic polypeptide is an additional incretin hormone that amplifies insulin production and controls glucose homeostasis [8]. Additionally, these hormones encourage the pancreas to produce insulin. They also slow down the stomach's emptying and reduce appetite, which helps to curb hunger and encourage weight reduction, and they decrease the synthesis of glucagon, a hormone that boosts blood sugar levels. Also, the synergistic pharmacological stimulation may enhance the functioning of β-cells. As a result, this combination treatment has become a popular option [9,10].

Tirzepatide was created in this situation as a new glucagon-like peptide one receptor agonist and glucose-dependent insulinotropic polypeptide receptor co-agonist that permits once-weekly subcutaneous delivery to help with blood sugar regulation and aids in weight loss [11,12]. It has been demonstrated that tirzepatide works on a glucagon-like peptide one receptor agonists, and glucose-dependent insulinotropic polypeptide which is an additional incretin hormone that amplifies insulin production and controls glucose homeostasis, and this makes it work better than other glucagon-like peptides one receptor agonists at lowering blood sugar and promoting weight reduction. Tirzepatide's efficiency in improving weight reduction, which is a major issue for many patients with type 2 diabetes and a risk factor for diabetes-related problems, is one of its main benefits [13]. In addition to its effects on blood sugar control and weight loss, tirzepatide may also improve cardiovascular health. Cardiovascular disease, which may cause heart attacks, strokes, and other serious problems, is more likely to occur in type 2 diabetics. There is still much to learn about the mechanism through which tirzepatide lowers the risk of cardiovascular disease. However, it is assumed that it is connected to its impacts on weight loss and blood sugar regulation, as well as its capacity to lower inflammation and enhance lipid metabolism [14,15]. The cost of tirzepatide is one of its limitations. Tirzepatide is likely to be costly and not be covered by all insurance policies, like many new drugs. This could make it difficult for certain patients, especially those without strong insurance coverage to get access to this drug [16].

Certain gastrointestinal manifestations, such as nausea, vomiting, diarrhea, and abdominal pain, have been reported with the use of tirzepatide [17-19]. However, there is a lack of a systematic analysis of the gastrointestinal manifestations and the use of tirzepatide. Since these manifestations are likely to affect the quality of life and may result in work-related absences, it is important to characterize the manifestations and their frequency. Therefore, we conducted a systematic review to assess the frequency of gastrointestinal manifestations and how seriously gastrointestinal issues affect people with type 2 diabetes on tirzepatide.

## Review

Materials and methods

In this systematic review, we used Preferred Reporting of Systematic Reviews and Meta-Analyses (PRISMA) 2020 guidelines [20].

Search Strategy and Selection Criteria

Articles were identified via MEDLINE (via PubMed), Web of Science, and Cochrane Library bibliographic databases using the search terms “Tirzepatide” and “Gastrointestinal Manifestations” and were restricted to randomized controlled trials. No restrictions were performed on language. The searches and data extractions were completed independently by two authors (A. Khan and M. Nouh) until May 1, 2023. Any disagreement in the literature screening or data extraction was resolved through discussion. The inclusion criteria of this study were based on PICOS criteria [21]: 1) Population: Any patients above 45 years with uncontrolled type 2 diabetes, who receive glucagon-like peptide one receptor agonist drugs, and have baseline HbA1C more than 7%. 2) Intervention/Comparison: Tirzepatide / Placebo, glucagon-like peptide one receptor agonists drugs, and insulin degludec. 3) Outcomes: incidence rate of gastrointestinal manifestations such as (nausea, constipation, decreased appetite, dyspepsia, diarrhea, and vomiting) of tirzepatide compared to placebo, glucagon-like peptide one receptor agonists drugs, and insulin degludec. 4) Study design: Randomized controlled trials. Duplicate studies and case reports were excluded from the study.

Outcomes, Data Analysis, and Risk of Bias

The primary outcome was gastrointestinal manifestations. The incidence of gastrointestinal manifestations in the tirzepatide arm was estimated and evaluated relative to the comparator (placebo, glucagon-like peptide one receptor agonists drugs, and insulin degludec) arm. Mantel-Haenszel risk ratios (RRs) with 95% confidence intervals (CIs) were assessed using random-effects models. A P < 0.05 was considered significant. Mantel-Haenszel method and heterogeneity test (I^2^) are provided in this study to show the percentage of variability in results across studies. We estimated the degree of heterogeneity among the trial results using the I^2^ statistics (25%, 50%, and 75% represented low, moderate, and high heterogeneity). To investigate potential causes of heterogeneity, subgroup analysis, and sensitivity analyses were performed. The quality of individual studies was assessed through the Cochrane Collaboration's tool for assessing the risk of bias. All these operations were implemented through Review Manager (RevMan) Version 5.4 software. RevMan is a software created by Cochrane Library for preparing and maintaining reviews that include language, research characteristics, comparison tables, and study data. It may do a meta-analysis of the provided data and graphically display the findings [22].

Data Extraction and Sensitivity Test

Eligible studies were reviewed, and the following data were extracted: (1) author name; (2) year of publication; (3) study location; (4) study design; (5) study title; (6) Patient Count; (7) Course Duration. Data extraction was performed independently by two reviewers (S. Alzahrani and A. Alshehri). Disagreements were resolved by a third reviewer (H. Karrar). In order to evaluate the effect of each study on the combined effect estimate, We performed a sensitivity analysis using a jackknife approach whereby each study is removed individually to test the robustness of the pooled HR with 95% CI. Also, the same approach was conducted to resolve the presence of any significant heterogeneity.

Result

Results of Literature Search

**Figure 1:**
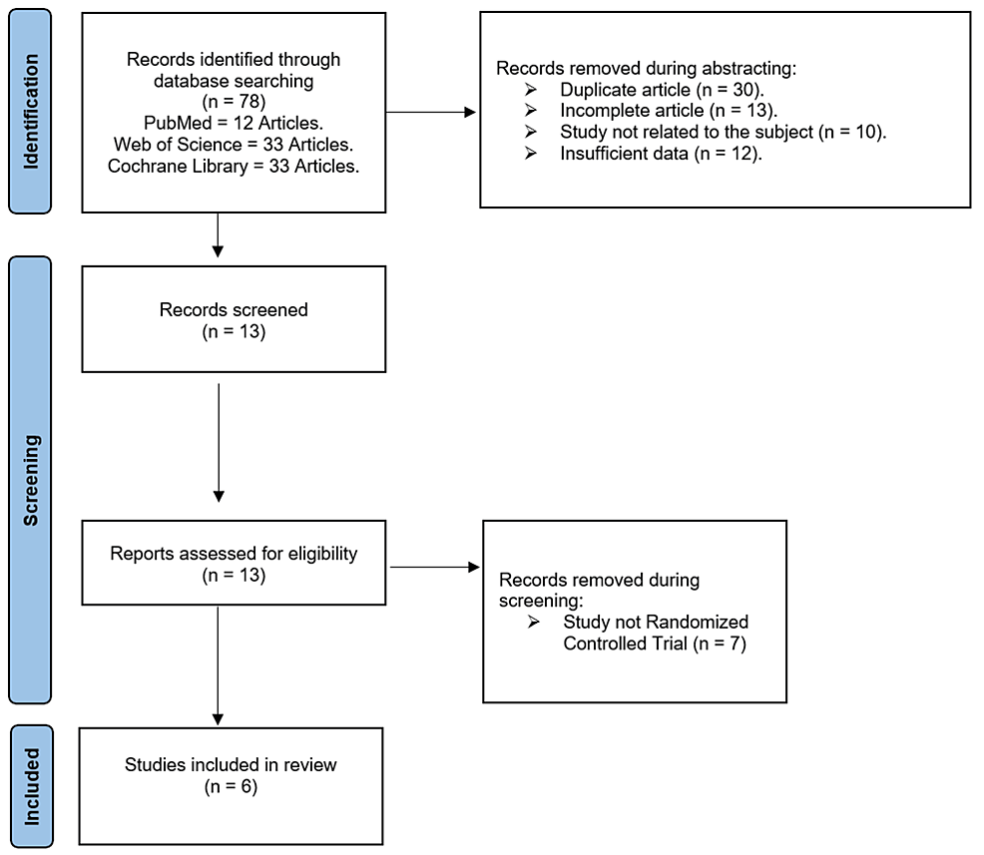
PRISMA flowchart. PRISMA: Preferred Reporting of Systematic Reviews and Meta-Analyses.

Seventy-eight suitable research studies were found in the initial literature search conducted using electronic web resources. The whole text of 13 papers was evaluated for potential inclusion after duplicates, incomplete articles, and insufficient data were excluded based on titles and abstracts. There were still six studies available for quantitative analysis. Figure 1 presents the literature search summary and findings.

Characteristics of Included Studies

In total, 4,586 patients were included in our analysis. Of these patients, 1,120 received a 5mg dose of tirzepatide, 1,120 received a 10mg dose of tirzepatide, 1,151 received a 15mg dose of tirzepatide, 312 received a placebo, 54 received a 1.5mg dose of dulaglutide, 469 received 1mg dose of semaglutide, and 360 received insulin degludec. All sex trials used the same overall doses of tirzepatide (5mg, 10mg, and 15mg weekly), while the comparators were placebo once weekly, glucagon-like peptide one receptor agonists drugs such as dulaglutide 1.5mg weekly, and semaglutide 1mg weekly, and insulin degludec 10U per day. Included trials were published between 2018 and 2023, and they were conducted in the USA (n = 5), India (n = 1), Japan (n = 2), Poland (n = 1), Slovakia (n = 1), Argentina (n = 1), Australia (n = 1), Mexico (n = 1), and Germany (n = 1). The duration of the treatment ranged from 12 to 52 weeks. The summary of the included studies and their main results are shown in Table 1; the baseline characteristics of their populations are shown in Table 2.

**Table 1:** Characteristics of included studies.

Author and year of publication	Study Design	Title	Location	Number of Patient	Course Duration
Julio Rosenstock 2021 [19]	Randomized Controlled Trials. Phase 3 trial. Multi-Center.	Efficacy and safety of a novel dual GIP and GLP-1 receptor agonist tirzepatide in patients with type 2 diabetes (SURPASS-1): a double-blind, randomised, phase 3 trial.	India, Japan, Mexico, and United States.	Tirzepatide 5mg: 121 Tirzepatide 10mg: 121 Tirzepatide 15mg: 121 Vs Placebo: 115	40 weeks
Dominik Dahl 2022 [23]	Randomized Controlled Trials, Double-blind. Phase 3 trial.	Effect of Subcutaneous Tirzepatide vs Placebo Added to Titrated Insulin Glargine on Glycemic Control in Patients With Type 2 Diabetes: The SURPASS-5 Randomized Clinical Trial.	Germany, Poland, Japan, and United States	Tirzepatide 5mg: 116 Tirzepatide 10mg: 119 Tirzepatide 15mg: 120 Vs Placebo: 120	40 weeks
Juan Pablo Frias 2021 [24]	Randomized Controlled Trials. Phase 3 trial.	Tirzepatide versus Semaglutide Once Weekly in Patients with Type 2 Diabetes.	128 sites in Australia, Argentina, and United States.	Tirzepatide 5mg: 470 Tirzepatide 10mg: 469 Tirzepatide 15mg: 470 Vs Semaglutide 1mg: 469	40 weeks
Bernhard Ludvik 2021 [25]	Randomized Controlled Trials. Phase 3 trial. Multi-Center.	Once-weekly tirzepatide versus once-daily insulin degludec as add-on to metformin with or without SGLT2 inhibitors in patients with type 2 diabetes (SURPASS-3): a randomised, open-label, parallel-group, phase 3 trial.	Argentina, Austria, Greece, Hungary, and Poland.	Tirzepatide 5mg: 358 Tirzepatide 10mg: 360 Tirzepatide 15mg: 359 Vs Insulin Degludec: 360	52 weeks
Juan Pablo Frias 2020 [26]	Randomized Controlled Trials, Double-blind. Phase 2 trial.	Efficacy and tolerability of tirzepatide, a dual glucose-dependent insulinotropic peptide and glucagon-like peptide-1 receptor agonist in patients with type 2 diabetes: A 12-week, randomized, double-blind, placebo-controlled study to evaluate different dose-escalation regimens.	United State	Tirzepatide 15mg: 28 Vs Placebo: 26	12 weeks
Juan Pablo Frias 2018 [27]	Randomized Controlled Trials. Phase 2 trial.	Efficacy and safety of LY3298176, a novel dual GIP and GLP-1 receptor agonist, in patients with type 2 diabetes: a randomised, placebo-controlled and active comparator-controlled phase 2 trial	47 sites in Poland, Puerto Rico, Slovakia, and United States	Tirzepatide 5mg: 55 Tirzepatide 10mg: 51 Tirzepatide 15mg: 53 Vs Dulaglutide 1.5mg: 54 Placebo: 51	26 weeks

**Table 2:** Patient demographics and baseline characteristics.

Author Name	Julio Rosenstock 2021 [19]		Dominik Dahl 2022 [23]		Juan Pablo Frias 2021 [24]		Bernhard Ludvik 2021 [25]		Juan Pablo Frias 2020 [26]		Juan Pablo Frias 2018 [27]		
	Tirzepatide	Placebo	Tirzepatide	Placebo	Tirzepatide	Semaglutide	Tirzepatide	Degludec	Tirzepatide	Placebo	Tirzepatide	Placebo	Dulaglutide
No. of patient	5mg: 121 10mg: 121 15mg: 121	115	5mg: 116 10mg: 119 15mg: 120	120	5mg: 470 10mg: 469 15mg: 470	469	5mg: 358 10mg: 360 15mg: 359	360	15mg: 28	26	5mg:55 10mg:51 15mg:53	51	54
Gender (Male)	5mg: 56 10mg: 72 15mg: 63	56	5mg: 61 10mg: 72 15mg: 65	66	5mg: 205 10mg: 238 15mg: 214	225	5mg: 200 10mg: 195 15mg: 194	213	15mg: 16	12	5mg:34 10mg:30 15mg:22	29	24
Gender (Female)	5mg: 65 10mg: 49 15mg: 58	59	5mg: 55 10mg: 47 15mg: 55	54	5mg: 265 10mg: 231 15mg: 256	244	5mg: 158 10mg: 165 15mg: 165	147	15mg:12	14	5mg:21 10mg:21 15mg:31	22	30
Mean Age	5mg: 54 10mg:56 15mg: 53	54	5mg: 62 10mg: 60 15mg: 61	60	5mg: 56 10mg: 57 15mg: 56	57	5mg: 57 10mg: 57 15mg: 57	58	15mg: 55	56	5mg:58 10mg:56 15mg:56	56	58
Baseline HbA1C	5mg:7.97 10mg:7.90 15mg: 7.85	8.05	5mg: 8.3 10mg: 8.4 15mg: 8.2	8.37	5mg: 8.32 10mg: 8.3 15mg: 8.3	8.25	5mg: 8.17 10mg: 8.18 15mg: 8.21	8.12	15mg: 8.5	8.2	5mg:8.2 10mg: 8.2 15mg: 8.1	8	8.1
Baseline BMI	5mg:32.2 10mg:32.2 15mg:31.5	31.7	5mg: 34 10mg: 33 15mg: 33	33.2	5mg: 33.8 10mg:34.3 15mg:34.5	34.2	5mg:33.6 10mg:33.4 15mg:33.7	33.4	15mg: 32	32.5	5mg:33 10mg:32 15mg: 32	32	32
Duration of Diabetes (years)	5mg: 4.6 10mg: 4.9 15mg: 4.8	4.5	5mg: 14 10mg: 13 15mg: 14	12.9	5mg:9.1 10mg:8.4 15mg:8.7	8.3	5mg:8.5 10mg:8.4 15mg: 8.5	8.1	15mg: 8.2	8.8	5mg: 8.9 10mg:7.9 15mg: 8.5	8.6	9.3

Quality of the Included Studies

**Figure 2:**
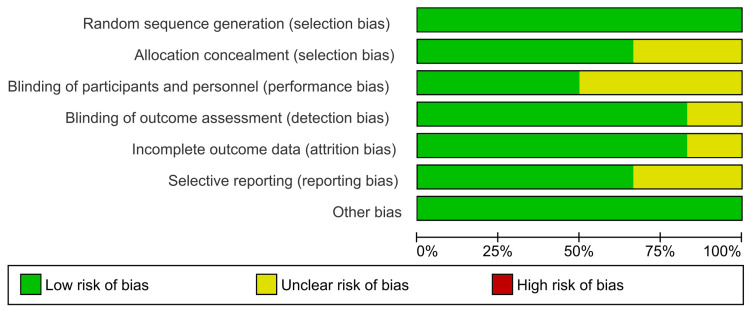
Risk of bias graph according to Cochrane Risk of Bias assessment tool.

**Figure 3:**
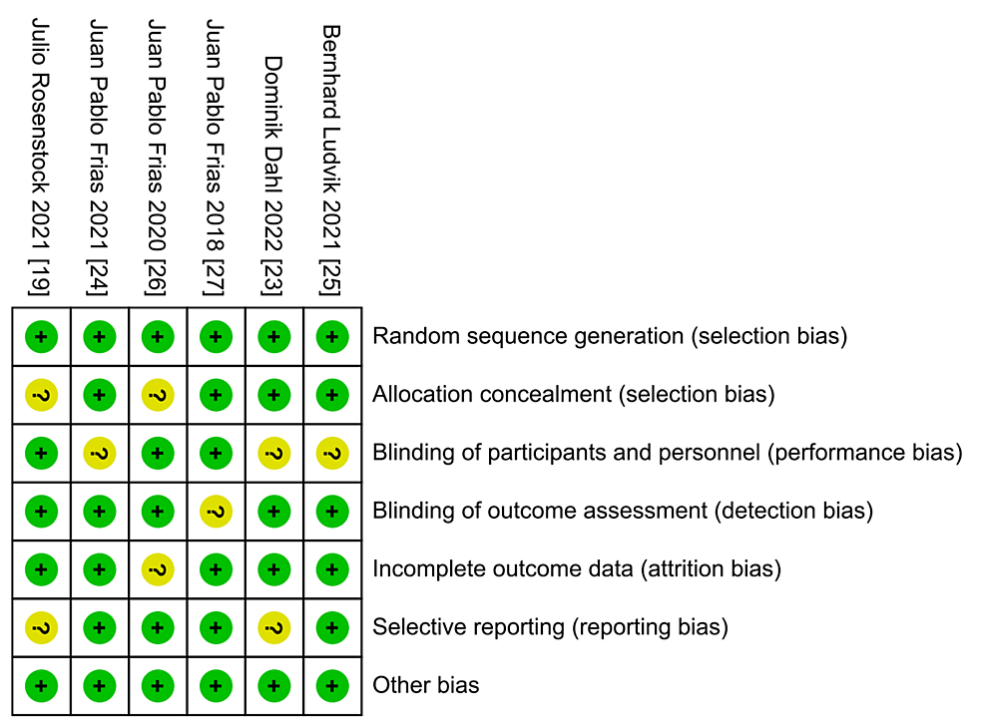
Risk of bias summary according to Cochrane Risk of Bias assessment tool.

According to The Cochrane Collaboration tool, the included trials ranged from low to moderate quality. The risk of bias graph of quality assessment domains of the included studies is shown in Figure 2, while the summary of quality assessment domains of the included studies is shown in Figure 3.

Overall Outcome

**Figure 4:**
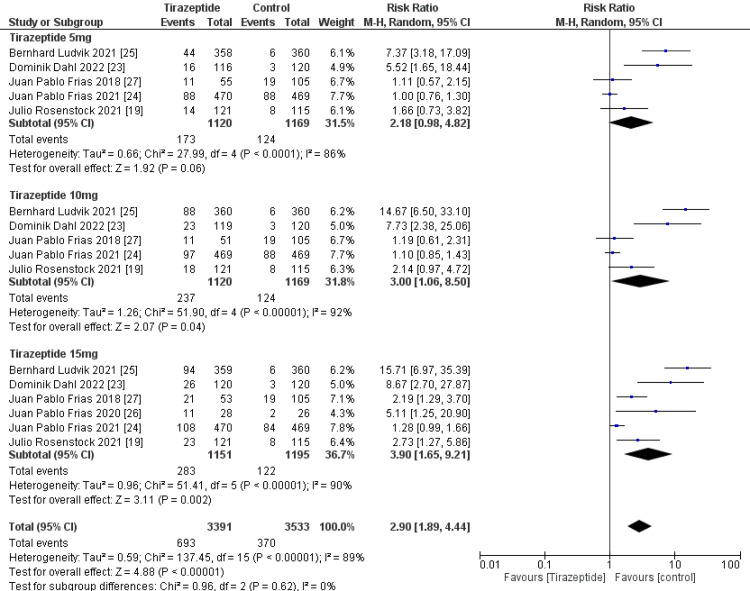
Forest plot showing the risk ratios of the incidence rate of nausea using random-effects models. A vertical line, “no difference” point between the two groups; horizontal line, 95% confidence interval; squares, risk ratios; diamonds, pooled risk ratios. CI, confidence interval; MH, Mantel-Haenszel.

**Figure 5:**
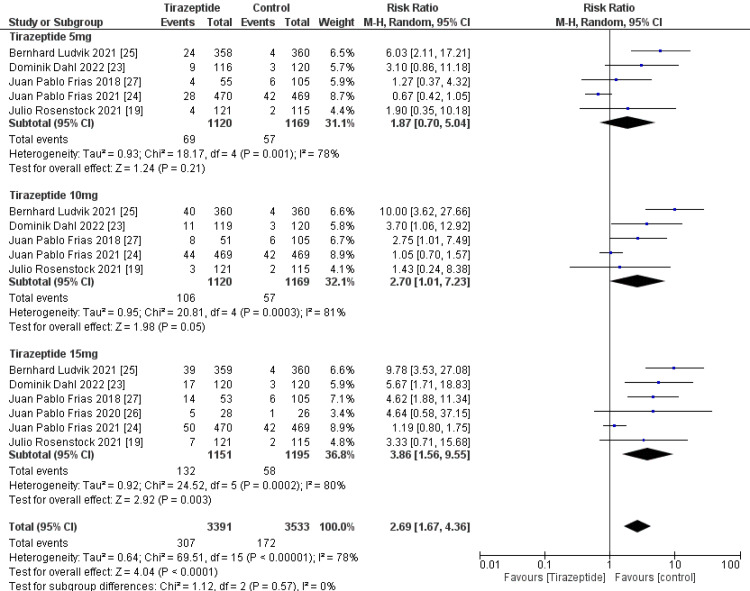
Forest plot showing the risk ratios of the incidence rate of vomiting using random-effects models. A vertical line, “no difference” point between the two groups; horizontal line, 95% confidence interval; squares, risk ratios; diamonds, pooled risk ratios. CI, confidence interval; MH, Mantel-Haenszel.

**Figure 6:**
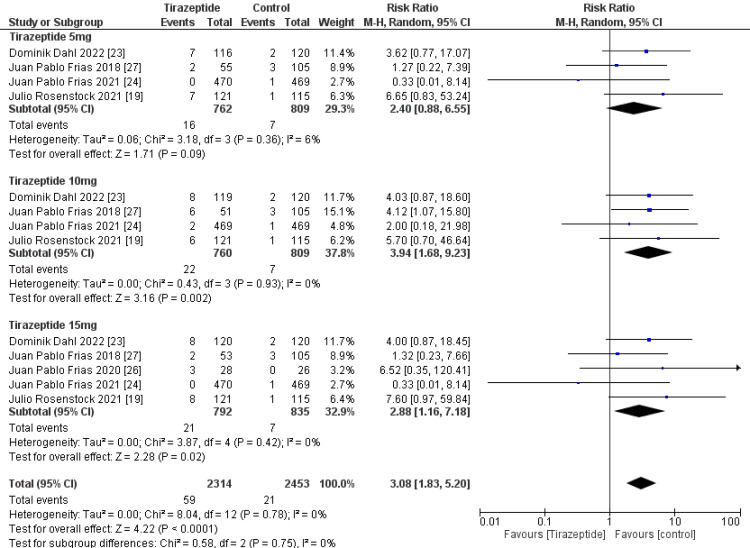
Forest plot showing the risk ratios of the incidence rate of constipation using random-effects models. A vertical line, “no difference” point between the two groups; horizontal line, 95% confidence interval; squares, risk ratios; diamonds, pooled risk ratios. CI, confidence interval; MH, Mantel-Haenszel.

**Figure 7:**
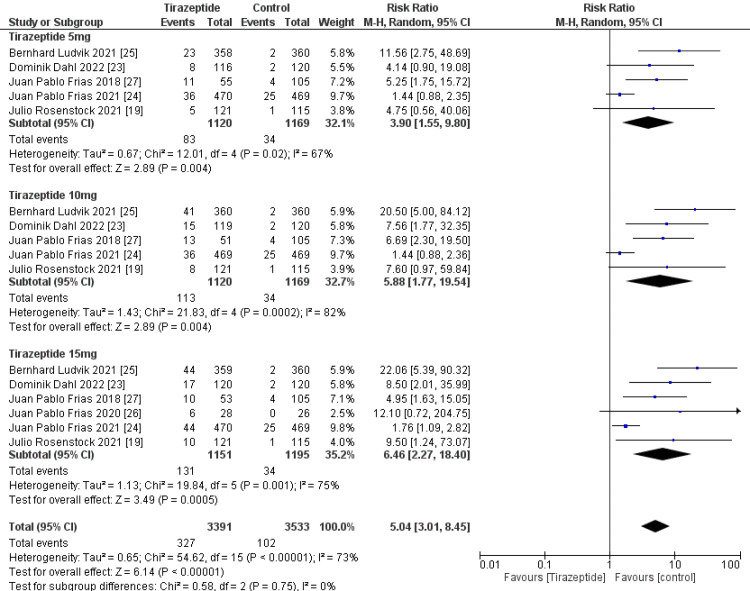
Forest plot showing the risk ratios of the incidence rate of decreased appetite using random-effects models. A vertical line, “no difference” point between the two groups; horizontal line, 95% confidence interval; squares, risk ratios; diamonds, pooled risk ratios. CI, confidence interval; MH, Mantel-Haenszel.

**Figure 8:**
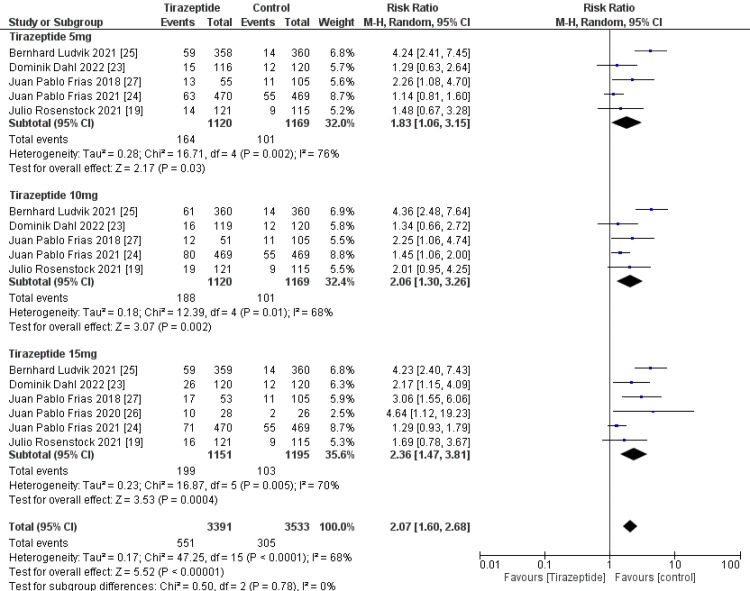
Forest plot showing the risk ratios of the incidence rate of diarrhea using random-effects models. A vertical line, “no difference” point between the two groups; horizontal line, 95% confidence interval; squares, risk ratios; diamonds, pooled risk ratios. CI, confidence interval; MH, Mantel-Haenszel.

**Figure 9:**
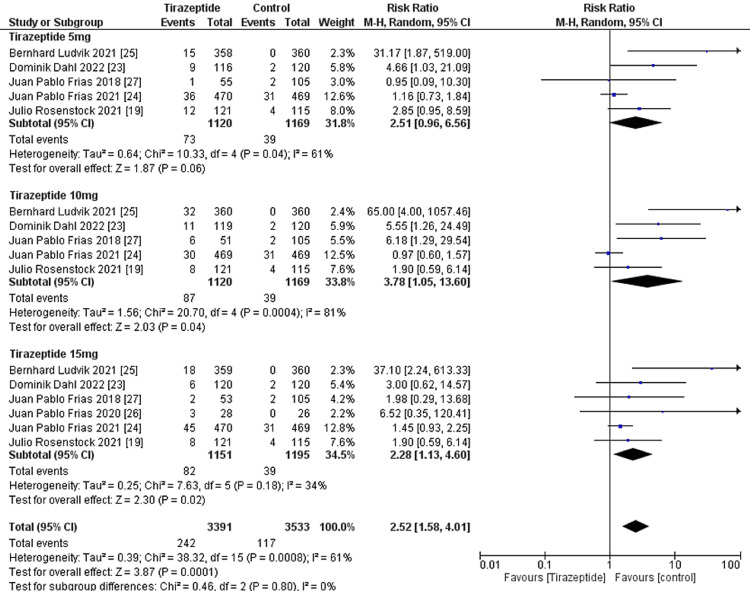
Forest plot showing the risk ratios of the incidence rate of dyspepsia using random-effects models. A vertical line, “no difference” point between the 2 groups; horizontal line, 95% confidence interval; squares, risk ratios; diamonds, pooled risk ratios. CI, confidence interval; MH, Mantel-Haenszel.

A total of 4,586 participants were involved in six randomized controlled trials. Gastrointestinal manifestations of the included studies are shown in Table 3. Tirzepatide was given to the majority of patients to control their blood sugar levels and aid in weight loss, and the comparators were placebo, glucagon-like peptide one receptor agonists drugs, and insulin degludec. The dose of tirzepatide was 5mg, 10mg, and 15mg weekly. The incidence rate of nausea in patients who receive tirzepatide was 20.43%, while the incidence rate in the comparators was 10.47%, and it was significantly higher in the tirzepatide arm than in the comparators as shown in Figure 4 (RR, 2.90; 95% CI, 1.89 to 4.44; P ≤ 0.00001). The incidence rate of vomiting in patients who receive tirzepatide was 9.05%, while the rate in the comparators was 4.86% and it was significantly higher in the tirzepatide arm than in the comparators as shown in Figure 5 (RR, 2.69; 95% CI, 1.67 to 4.36; P ≤ 0.0001). The incidence rate of constipation in patients who receive tirzepatide was 2.54%, while the rate in the comparators was 0.856%, and it was significantly higher in the tirzepatide arm than in the comparators as shown in Figure 6 (RR, 3.08; 95% CI, 1.83 to 5.20; P ≤ 0.0001). The incidence rate of decreased appetite in patients who receive tirzepatide was 9.64%, while the rate in the comparators was 2.88%, and it was significantly higher in the tirzepatide arm than in the comparators as shown in Figure 7 (RR, 5.04; 95% CI, 3.01 to 8.45; P ≤ 0.00001). The incidence rate of diarrhea in patients who receive tirzepatide was 16.24%, while the rate in the comparators was 8.63%, and it was significantly higher in the tirzepatide arm than in the comparators as shown in Figure 8 (RR, 2.07; 95% CI, 1.60 to 2.68; P ≤ 0.00001). The incidence rate of dyspepsia in patients who receive tirzepatide was 7.13%, while the rate in the comparators was 3.31%, and it was significantly higher in the tirzepatide arm than in the comparators as shown in Figure 9 (RR, 2.52; 95% CI, 1.58 to 4.01; P ≤ 0.0001). The incidence of most the gastrointestinal manifestations increases when the dose increases.

**Table 3:** Gastrointestinal manifestations of included studies.

Drug	Tirzepatide 5mg (n = 1120)	Tirzepatide 10mg (n = 1120)	Tirzepatide 15mg (n = 1151)	Placebo (n = 312)	Dulaglutide 1.5mg (n = 54)	Semaglutide 1mg (n = 469)	Insulin Degludec 10U (n = 360)
Nausea	173	237	283	13	16	88	6
Vomiting	69	106	132	5	7	42	4
Dyspepsia	73	87	82	6	2	31	0
Decrease Appetite	83	113	131	4	3	25	2
Diarrhea	164	188	199	27	9	55	14
Constipation	16	22	21	2	4	28	0

Sensitivity Test

We performed sensitivity analysis using a jackknife approach whereby each study is removed individually to test the robustness of the pooled HR with 95% CI. As shown in Table 4. The variability of heterogeneity in the studies may be due to the variable definitions of gastrointestinal manifestation, ranges of doses used, range of drugs used, and variability of duration of diabetes.

**Table 4:** Sensitivity analysis.

Study	Tirzepatide 5mg		Tirzepatide 10mg			Tirzepatide 15mg		
	RR (95% CI)	I^2^	RR (95% CI)		I^2^	RR (95% CI)		I^2^
Nausea								
Julio Rosenstock 2021 [19]	2.38 [0.87, 6.47]	89%	3.31 [0.88, 12.5]		94%	4.27 [1.51, 12.1]		92%
Dominik Dahl 2022 [23]	1.82 [0.80, 4.12]	86%	2.45 [0.80, 7.47]		93%	3.39 [1.38, 8.35]		91%
Juan Pablo Frias 2021 [24]	2.81 [1.05, 7.49]	81%	4.01 [1.10, 14.6]		90%	5.1 [2.05, 12.68]		82%
Bernhard Ludvik 2021 [25]	1.46 [0.83, 2.55]	65%	1.82 [0.94, 3.51]		76%	2.65 [1.43, 4.92]		77%
Juan Pablo Frias 2020 [26]	-	-	-		-	3.76 [1.47, 9.58]		92%
Juan Pablo Frias 2018 [27]	2.69 [0.91, 7.99]	89%	3.88 [0.49, 16.0]		94%	4.57 [1.38, 15.1]		92%
Vomiting								
Julio Rosenstock 2021 [19]	1.89 [0.59, 6.00]	83%	3.03 [0.97, 9.46]		86%	3.99 [1.42, 11.2]		83%
Dominik Dahl 2022 [23]	1.68 [0.53, 5.30]	81%	2.52 [0.78, 8.15]		84%	3.60 [1.29, 10.0]		81%
Juan Pablo Frias 2021 [24]	2.82 [1.36, 5.86]	25%	3.94 [1.77, 8.80]		43%	5.68 [3.34, 9.65]		0%
Bernhard Ludvik 2021 [25]	1.24 [0.57, 2.70]	52%	1.76 [0.88, 3.49]		49%	3.04 [1.29, 7.17]		71%
Juan Pablo Frias 2020 [26]	-	-	-		-	3.80 [1.41, 10.2]		83%
Juan Pablo Frias 2018 [27]	2.10 [0.60, 7.38]	83%	2.72 [0.75, 9.80]		85%	3.76 [1.25, 11.4]		81%
Constipation								
Julio Rosenstock 2021 [19]	1.83 [0.61, 5.46]	0%	3.66 [1.45, 9.29]		0%	2.28 [0.82, 6.30]		0%
Dominik Dahl 2022 [23]	1.80 [0.40, 8.19]	28%	3.90 [1.40, 10.9]		0%	2.41 [0.67, 8.69]		18%
Juan Pablo Frias 2021 [24]	2.95 [1.07, 8.16]	0%	4.35 [1.75, 10.8]		0%	3.49 [1.35, 9.04]		0%
Juan Pablo Frias 2020 [26]	-	-	-		-	2.57 [0.89, 7.46]		15%
Juan Pablo Frias 2018 [27]	3.05 [0.81, 11.4]	18%	3.83 [1.28, 11.4]		0%	3.84 [1.32, 11.2]		0%
Decreased Appetite								
Julio Rosenstock 2021 [19]	3.86 [1.36, 11.0]	74%	5.68 [1.46, 22.1]		86%	6.16 [1.94, 19.6]		78%
Dominik Dahl 2022 [23]	3.97 [1.30, 12.1]	74%	5.63 [1.35, 23.5]		85%	6.22 [1.85, 20.9]		77%
Juan Pablo Frias 2021 [24]	5.99 [2.93, 12.2]	0%	9.14 [4.57, 18.3]		0%	8.96 [4.54, 17.7]		0%
Bernhard Ludvik 2021 [25]	2.84 [1.24, 6.50]	52%	4.15 [1.38, 12.5]		74%	4.46 [1.82, 11.0]		58%
Juan Pablo Frias 2020 [26]	-	-	-		-	6.11 [2.00, 18.7]		79%
Juan Pablo Frias 2018 [27]	3.70 [1.17, 11.7]	69%	5.88 [1.22, 28.4]		84%	7.27 [1.85, 28.6]		79%
Diarrhea								
Julio Rosenstock 2021 [19]	1.92 [0.99, 3.72]	82%	2.08 [1.18, 3.65]		76%	2.54 [1.45, 4.48]		76%
Dominik Dahl 2022 [23]	1.98 [1.01, 3.89]	82%	2.27 [1.31, 3.94]		74%	2.45 [1.35, 4.41]		76%
Juan Pablo Frias 2021 [24]	2.14 [1.20, 3.83]	64%	2.34 [1.37, 3.99]		59%	2.85 [1.99, 4.08]		18%
Bernhard Ludvik 2021 [25]	1.31 [1.00, 1.71]	0%	1.57 [1.22, 2.03]		0%	1.97 [1.30, 3.00]		50%
Juan Pablo Frias 2020 [26]	-	-	-		-	2.23 [1.36, 3.67]		74%
Juan Pablo Frias 2018 [27]	1.74 [0.90, 3.38]	81%	2.03 [1.16, 3.55]		75%	2.26 [1.30, 3.92]		73%
Dyspepsia								
Julio Rosenstock 2021 [19]	2.68 [0.68, 10.5]	67%	5.20 [0.85, 31.8]		86%	2.86 [1.04, 7.87]		48%
Dominik Dahl 2022 [23]	2.18 [0.73, 6.56]	63%	3.55 [0.78, 16.2]		83%	2.35 [0.99, 5.57]		44%
Juan Pablo Frias 2021 [24]	3.69 [1.30, 10.4]	30%	5.81 [1.57, 21.6]		62%	3.26 [1.26, 8.47]		24%
Bernhard Ludvik 2021 [25]	1.79 [0.89, 3.63]	38%	2.34 [0.88, 6.21]		68%	1.62 [1.10, 2.38]		0%
Juan Pablo Frias 2020 [26]	-	-	-		-	2.19 [1.04, 4.60]		41%
Juan Pablo Frias 2018 [27]	2.98 [0.98, 9.08]	71%	3.46 [0.77, 15.6]		83%	2.54 [1.08, 5.97]		48%

Discussion

Our meta-analysis of randomized controlled trials confirms a high incidence rate of gastrointestinal manifestations related to the use of tirzepatide. In our analysis, the mean age of patients in most included studies was above 55 years of age and the overall percentage of Baseline HbA1C was above 8. In most studies, the duration of treatment was above 40 weeks [19,23-25], while in two studies was below 40 weeks [26,27].

The incidence rate of nausea in patients who received tirzepatide was 20.43%, while the incidence rate in patients who received comparators was 10.47%. The incidence rate of vomiting in patients who received tirzepatide was 9.05%, while the incidence rate in patients who received comparators was 4.86%. The incidence rate of constipation in patients who received tirzepatide was 2.54%, while the incidence rate in patients who received comparators was 0.85%. The incidence rate of decreased appetite in patients who received tirzepatide was 9.64%, while the incidence rate in patients who received comparators was 2.88%. The incidence rate of diarrhea in patients who received tirzepatide was 16.24%, while the incidence rate in patients who received comparators was 8.63%. The incidence rate of dyspepsia in patients who received tirzepatide was 7.13%, while the incidence rate in patients who received comparators was 3.31%.

Several limitations related to this analysis are worthy of consideration. The studies in this analysis used variable definitions of gastrointestinal manifestations, which can make clinical application challenging. However, all definitions incorporated an element of nausea, vomiting, diarrhea, decreased appetite, dyspepsia, and constipation.

There are several strengths to our analysis. We only included studies that were randomized. In addition, tirzepatide dosing in the five included studies [19,23-25,27] were consistent (5mg, 10mg, and 15mg). The majority of the population was representative of patients from more than 10 continents, increasing the external validity of this meta-analysis. Finally, given that tirzepatide recipients frequently are complicated patients with type 2 diabetes, with a baseline HbA1C of more than 8 and Baseline BMI of more than 30. This analysis could be expected to be representative of other likely candidates for tirzepatide therapy in a real-world setting.

## Conclusions

Tirzepatide usage is linked to a significant prevalence of gastrointestinal symptoms, including nausea, constipation, decreased appetite, dyspepsia, diarrhea, and vomiting, in people with type 2 diabetes. These findings may influence clinical decision-making and patient counseling on the use of tirzepatide and have significant implications for the medication's tolerance and efficacy. Lifestyle modifications such as taking rest and drinking plenty of healthy liquids, eating easily digestible foods, avoiding spices, fried foods, alcohol, and other foods that are gastric irritants, and taking prescribed medications to treat the symptoms may decrease gastrointestinal symptoms. More research is required to evaluate lifestyle modifications and medications to reduce these negative effects and improve therapy for type 2 diabetic patients.

## References

[1] DeFronzo RA, Ferrannini E, Groop L (2015). Type 2 diabetes mellitus. Nat Rev Dis Primers.

[2] Ferrannini E, Cushman WC (2012). Diabetes and hypertension: the bad companions. Lancet.

[3] Saeedi P, Petersohn I, Salpea P (2019). Global and regional diabetes prevalence estimates for 2019 and projections for 2030 and 2045: Results from the International Diabetes Federation Diabetes Atlas, 9(th) edition. Diabetes Res Clin Pract.

[4] Malone JI, Hansen BC (2019). Does obesity cause type 2 diabetes mellitus (T2DM)? or is it the opposite?. Pediatr Diabetes.

[5] Lavie CJ, Milani RV, Ventura HO (2009). Obesity and cardiovascular disease: risk factor, paradox, and impact of weight loss. J Am Coll Cardiol.

[6] Min T, Bain SC (2021). The role of tirzepatide, dual GIP and GLP-1 receptor agonist, in the management of type 2 diabetes: the surpass clinical trials. Diabetes Ther.

[7] ElSayed NA, Aleppo G, Aroda VR (2023). 9. Pharmacologic approaches to glycemic treatment: standards of care in diabetes-2023. Diabetes Care.

[8] Nauck MA, Meier JJ (2016). The incretin effect in healthy individuals and those with type 2 diabetes: physiology, pathophysiology, and response to therapeutic interventions. Lancet Diabetes Endocrinol.

[9] Bastin M, Andreelli F (2019). Dual GIP-GLP1-receptor agonists in the treatment of type 2 diabetes: a short review on emerging data and therapeutic potential. Diabetes Metab Syndr Obes.

[10] Papachristou S, Popovic DS, Papanas N (2021). The new dual gastric inhibitory peptide/glucagon-like peptide 1 agonist tirzepatide in type 2 diabetes: Is the future bright?. Diabetes Metab Res Rev.

[11] Jastreboff AM, Aronne LJ, Ahmad NN (2022). Tirzepatide once weekly for the treatment of obesity. N Engl J Med.

[12] Thomas MK, Nikooienejad A, Bray R (2021). Dual GIP and GLP-1 receptor agonist tirzepatide improves beta-cell function and insulin sensitivity in type 2 diabetes. J Clin Endocrinol Metab.

[13] Karagiannis T, Avgerinos I, Liakos A, Del Prato S, Matthews DR, Tsapas A, Bekiari E (2022). Management of type 2 diabetes with the dual GIP/GLP-1 receptor agonist tirzepatide: a systematic review and meta-analysis. Diabetologia.

[14] Copur S, Tanriover C, Yavuz F, Tuttle KR, Kanbay M (2023). Tirzepatide and potential use for metabolically healthy obesity. Eur J Intern Med.

[15] Sattar N, McGuire DK, Pavo I, Weerakkody GJ, Nishiyama H, Wiese RJ, Zoungas S (2022). Tirzepatide cardiovascular event risk assessment: a pre-specified meta-analysis. Nat Med.

[16] Zhang X, McAdam Marx C (2023). Short-term cost-effectiveness analysis of tirzepatide for the treatment of type 2 diabetes in the United States. J Manag Care Spec Pharm.

[17] Kushner P, Anderson JE, Simon J, Boye KS, Ranta K, Torcello-Gómez A, Levine JA (2023). Efficacy and safety of tirzepatide in adults with type 2 diabetes: a perspective for primary care providers. Clin Diabetes.

[18] Borner T, Tinsley IC, Doyle RP, Hayes MR, De Jonghe BC (2022). Glucagon-like peptide-1 in diabetes care: can glycaemic control be achieved without nausea and vomiting?. Br J Pharmacol.

[19] Rosenstock J, Wysham C, Frías JP (2021). Efficacy and safety of a novel dual GIP and GLP-1 receptor agonist tirzepatide in patients with type 2 diabetes (SURPASS- 1): a double-blind, randomised, phase 3 trial. Lancet.

[20] Page MJ, McKenzie JE, Bossuyt PM (2021). The PRISMA 2020 statement: an updated guideline for reporting systematic reviews. BMJ.

[21] Major MP, Major PW, Flores-Mir C (2007). Benchmarking of reported search and selection methods of systematic reviews by dental speciality. Evid Based Dent.

[22] Van der Mierden S, Tsaioun K, Bleich A, Leenaars CH (2019). Software tools for literature screening in systematic reviews in biomedical research. ALTEX.

[23] Dahl D, Onishi Y, Norwood P, Huh R, Bray R, Patel H, Rodríguez Á (2022). Effect of subcutaneous tirzepatide vs placebo added to titrated insulin glargine on glycemic control in patients with type 2 diabetes: the surpass-5 randomized clinical trial. JAMA.

[24] Frías JP, Davies MJ, Rosenstock J (2021). Tirzepatide versus semaglutide once weekly in patients with type 2 diabetes. N Engl J Med.

[25] Ludvik B, Giorgino F, Jódar E (2021). Once-weekly tirzepatide versus once-daily insulin degludec as add-on to metformin with or without SGLT2 inhibitors in patients with type 2 diabetes (SURPASS- 3): a randomised, open-label, parallel-group, phase 3 trial. Lancet.

[26] Frias JP, Nauck MA, Van J (2020). Efficacy and tolerability of tirzepatide, a dual glucose-dependent insulinotropic peptide and glucagon-like peptide-1 receptor agonist in patients with type 2 diabetes: A 12-week, randomized, double-blind, placebo-controlled study to evaluate different dose-escalation regimens. Diabetes Obes Metab.

[27] Frias JP, Nauck MA, Van J (2018). Efficacy and safety of LY3298176, a novel dual GIP and GLP-1 receptor agonist, in patients with type 2 diabetes: a randomised, placebo-controlled and active comparator-controlled phase 2 trial. Lancet.

